# Infant Mental Health Research in Africa: a call for action for research in the next 10 years

**DOI:** 10.1017/gmh.2015.4

**Published:** 2015-06-02

**Authors:** Mark Tomlinson, Barak Morgan

**Affiliations:** 1 Department of Psychology, Stellenbosch University, Cape Town, Matieland, Stellenbosch, South Africa; 2 Global Risk Governance Program, Department of Public Law, University of Cape Town, Cape Town, South Africa

**Keywords:** Africa, call for action, child development, infant mental health

## Abstract

**Background.:**

Less than 3% of articles published in the peer reviewed literature include data from low- and middle-income countries – where 90% of the world's infants live.

**Methods.:**

In this paper, we discuss the context of infancy in Africa and the conditions of adversity obtaining in Africa.

**Results.:**

We discuss the implications of poverty on parenting, and linked to this outline the impact of maternal depression on infant development.

**Conclusions.:**

We outline three features of the field of infant mental health research in Africa, and issue a call for action about what we believe is needed in order to develop the field in the next decade.

## Introduction

At first blush – at least in the public imagination – ‘infant^†^^1^ mental health’ is not an easy concept to understand. How can an infant get depressed? Is there such a thing as an anxious infant? One of the founding fathers of psychology, William James, described the infant's world as a ‘blooming, buzzing confusion’ (p. 488) (James, 1890). As late as the 1940s there were still doubts about whether infants could distinguish colours and whether they could see faces. These false beliefs have been replaced by a wealth of data detailing how (for example) infants are immaculately attuned to the emotions of others (Trevarthen & Aitken, 2001), have basic numeracy skills (Van de Rijt & Van Luit, 1999) and are co-creators of relationships with caregivers and others (Nagy *et al*. 2010). The notion of infants being fundamentally in relation with another is aptly formulated by British paediatrician and psychoanalyst Winnicott when he stated ‘There is no such thing as an infant… without maternal care one would find no infant’ (p. 39) (Winnicott, 1966). The extreme dependency of infants on other people for their survival immediately places the infant (as well as the child) in relationship with others. From an evolutionary perspective it would therefore make sense that infants are born with the potential for sophisticated social, gestural and imitative interaction in order to ensure their own survival, and equally are vulnerable to mental health difficulties.

More recently, with the development of sophisticated brain measurement techniques and technologies, we now know the extent to which early experiences (even prenatally) are crucial in brain development (Webb *et al*. 2001). While there is considerable data detailing the development of infants and children in high-income countries (HIC), the same is not true for evidence on the diverse experiences and conditions that promote or impede infant development in low- and middle-income countries (LMIC), and more specifically in Africa. In this paper, we discuss the context of infancy in Africa, the implications of poverty on parenting, and linked to this provide a brief outline of the impact of maternal depression on infant and child development. We will then outline three priorities for research on infant mental health and infant development in Africa.

## Infants in Africa

In the global popular imagination Africa is frequently conceptualised in single country terms – people are referred to as African whether they come from Botswana or Egypt. In fact, Africa has a population of over a billion people, and high levels of diversity with up to 3000 languages and 54 states. Ethiopia alone has five religions and over 80 ethnic groups, while there are over 500 ethnic groups and 522 languages in Nigeria. This diversity should present fertile research ground – particularly in human development. The reality, however, is one of limited research outputs – the result of poor research infrastructure, lack of human resources, coupled with endemic poverty.

Africa is the poorest continent and has 16 of the top 20 poorest nations in the world, and is characterised by extremely high levels of infant mortality and morbidity. Globally, significant gains have been made in infant and child survival in the last two decades, with a child born today having half the risk of dying than if they were born in 1990 (Liu *et al*. 2014). Unfortunately, this is not true for Africa – Africa's share of child deaths has in fact increased since 1990. More than 50% of maternal and child deaths now occur in Africa although Africa comprises only 14% of the world's population (Lawn *et al*. 2014). Approximately one in eight children under the age of five continues to die in sub-Saharan Africa; with 121 deaths per 1000 live births. Poverty and lack of education are the key factors determining under-five mortality rates, manifesting as high rates of teenage pregnancies, low contraceptive use and lack of access to skilled birth attendance and undernourishment among children (Barros *et al*. 2012).

Despite the high levels of mortality, the African countries in which children are dying are the same ones in which many more children are surviving (Irwin *et al*. 2007). Approximately 200 million children in developing countries do not achieve their developmental and cognitive potential because of living in conditions of chronic poverty and their compounding lack of education (Grantham-McGregor *et al*. 2007). Lack of resources, particularly in early child development services impairs potential in children (Shonkoff *et al*. 2012).

## Parenting in contexts of poverty

Parenting is particularly sensitive to perturbations in the psychosocial context (Tomlinson, 2010). From a public health perspective, it has been argued that parenting difficulties are the proximal mechanism by which broader structural and societal factors operate (Link & Phelan, 1995). Another way of describing this is to conceptualise socio-economic status as an upstream determinant, while disease and parenting are downstream determinants (Gehlert *et al*. 2008). There are three predominant influences on parental functioning – parental factors and the personal psychological resources of the caregiver; child characteristics such as temperament and gender; and finally contextual conditions such as poverty, stress and support (Belsky, 1984). Parenting is embedded in a myriad of social factors that may affect child development (O'Connor & Scott, 2007). An important premise of this model is that there is a potentially inexorable co-variation of risk factors in the environment of any child (O'Connor & Scott, 2007). The seminal work of Sameroff has shown the limitations of considering only single risk factors in infant and child development (Sameroff & Seifer, 1983). It is the severity and chronicity of cumulative risk factors combined with persistent poor environmental conditions such as poverty and family instability that are more important than any single risk factor (Sameroff & Seifer, 1983).

Poverty and living in a violent community impact on the choices parents make to protect their children. For example, attempts at increased monitoring in order to protect the child from dangerous environments or from associating with an ‘inappropriate peer group’ often result in the escalation of the harsh and coercive aspects of caregiving (Beyers *et al*. 2003). In this scenario, poverty exerts its influence on infant and child outcome in a distal way via parenting practices. Poverty may also contribute to familial stress and maternal depression which in turn affects parenting, and by extension child outcome. Biological factors such as nutrition and intrauterine growth restriction may affect outcome in a proximal way (increased mortality and morbidity), but also in a distal way by increasing the vulnerability of the infant to an already stressed and adverse environment (Tomlinson, 2010). Factors such as foetal alcohol syndrome may result in mental impairment which may result in heightened levels of abuse and neglect. Parenting is rendered more difficult with an infant who has foetal alcohol syndrome, but it is the mental impairment that compromises parenting practices over and above the more immediate direct effects of poverty.

High-risk environments characteristic of much of Africa, and where community relationships may be less positive, lead to more stressful day-to-day interactions for families (Kochanska, 1997), resulting in parents that are depleted and unable to muster the necessary energy to parent (Halpern, 1993). Ghate and Hazel have argued that while many parents do well (even those living in poverty), parenting in environments characterised by poverty is more ‘risky’ than parenting elsewhere, and that the poorer the environment the more difficult parenting becomes (Garbarino & Kostelny, 1992).

## Maternal depression

In conditions of extreme poverty and instability, the demands on parents differ markedly from those facing parents in communities that have typically been the focus of research in infant mental health. Preoccupation with external problems (e.g. poverty and lack of partner support), as well as more immediate difficulties (e.g. trauma and losses), may directly affect the parent's capacity to be responsive to their infant. This difficulty may be further compounded by maternal mental health problems, in particular, by the occurrence of depression. Warm, sensitive and non-intrusive caregiving helps infants to regulate their behaviour and emotions. Disruptions such as maternal depression, absence or illness, may also however impact on infant and child development. Accumulating evidence suggests that maternal mental health may be contributing to a failure to improve key public health outcomes for women and infants (Rahman *et al*. 2013). For the purposes of this paper, a powerful case could be made for focusing on other important influences on infant mental health such as physical maltreatment or psychological control. However, given the increasing global burden of disease attributable to depression (Lim *et al*. 2012), coupled with the fact that depression is a multigenerational disorder, (England & Sim, 2009), we have decided to focus on maternal depression in this paper.

In South Africa, up to 35%–47% of women have been diagnosed with major depressive disorder (Cooper *et al*. 1999; Rochat *et al*. 2011; Tsai & Tomlinson, 2012). Not only is major depressive disorder accompanied by substantial morbidity and disability (Lim *et al*. 2012), but also new-born children of mothers with depression have poorer health (Rahman *et al*. 2004) and socio-emotional development (Weissman *et al*. 2006), with adverse implications for their psychosocial, cognitive and economic outcomes (Heckman, 2007). Maternal depression is a public health concern in and of itself, but also has intergenerational effects due to its association with disturbances in the mother–infant relationship, poor new-born health (Gavin *et al*. 2005) and poorer psychosocial, cognitive and economic outcomes (Heckman, 2007). A caregiver's responsiveness to the attachment needs of a child has been shown to be strongly associated with whether the child develops a secure or insecure attachment (Tomlinson *et al*. 2005). A secure attachment in turn benefits the child in terms of lower chances of behavioural problems, improved social functioning, better relationships with their peers and enhanced school performance (van Ijzendoorn *et al*. 1995, Young *et al*. 2002). All of these caregiver tasks may be compromised in the contexts of poverty and high adversity, and certainly in the case of maternal depression.

Having briefly sketched some of the demands on parents in contexts of poverty, we will now outline three features of the field of infant mental health research in Africa, and linked to each of these issues a call for action for research in the next 10 years.

### State of the knowledge base

#### Current context

Over 90% of the world's infants are born in LMIC (Population Reference Bureau, 2013). The so-called ‘10/90 gap’ (Saxena *et al*. 2006), refers to the fact that only 10% of the worldwide spending on health research is directed towards the problems that primarily affect the poorest 90% of the world's population. A study conducted in 2003 surveying articles on infancy between 1996 and 2001 from major international journals, reported that a meagre 5% of articles emanated from parts of the world other than North America, Europe or Australasia (Tomlinson & Swartz, 2003). In a follow-up study reviewing articles published between 2002 and 2012 only 2.3% of articles published in 11 years included data from LMIC – where 90% of the world's infants live (Tomlinson *et al*. 2014). The knowledge that we do have about infant mental health from Africa and other LMIC is predominantly from studies with small sample sizes and in a single setting (Walker *et al*. 2007). This narrow participant database and restricted range continues to constrain our understanding of infant mental health, and impedes our ability to begin untangling the many idiosyncrasies of infant development and caregiving (Bornstein *et al*. 2012), and affects our ability to identify which domains of development are susceptible to which experiences.

#### Call for action: increased funding for research and research capacity development in Africa

First and foremost, in order to deepen the knowledge base on infant mental health in Africa, there needs to be a substantial increase in research funding. While significant funding for mental health research broadly (not simply infant mental health) has not been forthcoming from funders, there have been a number of recent initiatives from Grand Challenges Canada, the Department for International Development (UK Aid) and the National Institute of Mental Health (USA) that have injected significant funding into mental health research. Much of this funding however is targeted at adult mental health research. A notable exception is that of the Grand Challenges Canada Saving Brains initiative that has provided funding to 11 studies in LMIC for the follow-up of cohorts that received an intervention in the first 1000 days with the aim of increasing the evidence base about child cognitive development.

Linked to the need for increased funding, concerted efforts need to be made to improve research capacity throughout Africa. Initiatives such as the Centre for Public Mental Health (joint initiative between Stellenbosch University and the University of Cape Town in South Africa) that offers a distance M. Phil. degree for applicants throughout Africa is an example of one such initiative. Other multi-country research studies such as the Programme for Improving Mental Health Care which is being conducted in three African countries (Ethiopia, South Africa and Uganda) have large components of capacity building (with six Ph. D. students in Ethiopia alone) will also contribute to improving research capacity on the continent. Increased resources for research, and focus on capacity building in Africa are essential if the 10/90 gap is to be meaningfully reversed.

### Longitudinal and early intervention research

#### Current context

To the best of our knowledge there is only one longitudinal African study (tracking infant and child development) that includes data on at least three generations (original mother, child and then child of the child) – Birth to Twenty Study (Richter *et al*. 2004). As has already been shown, data on infant mental health and development from Africa are limited, and that which exist are largely cross-sectional in nature. Longitudinal studies in Africa are essential if we are to be in a position to make causal attributions across the lifespan. Increasing investment in longitudinal studies in a variety of contexts in Africa will allow us to begin unravelling the dynamics of infant and child development across the life cycle, and allow us to move from complicated correlations to developing causal models of prediction in one of the most diverse continents on earth (Butz & Torrey, 2006).

In addition, while the Birth to Twenty has produced seminal data on infant and child development across the lifespan (and will continue to do so), it is epidemiological and did not include an intervention (early or late). Given the adversity that characterises much of infant and child development in Africa, exploring the long-term impact of interventions across the lifespan using gold standard randomised controlled trials is essential.

#### Call for action: increased funding for longitudinal and early intervention research

We call for increased investment in longitudinal research as well as early intervention and prevention programmes, to improve infant mental health and optimal infant and child development. We need to establish an evidence-base from LMIC to identify the most promising interventions specifically for infant and child mental health. While it is tempting to translate evidence from HIC to LMIC, studies need to be replicated in specific contexts to establish their relevance for these settings (Klasen, 2013). Multinational research such as this, constructed within a sensitive contextual framework, will allow the developmental community to distinguish how children's experiences in different settings shape specific trajectories of their development (Bornstein *et al*. 2012).

There is emerging longitudinal evidence from the continent about infant and child development across the lifespan and in the context of an intervention taking place in the first 1000 days. Cooper *et al.* designed a manualised home-based intervention delivered by community health workers in a peri-urban settlement in South Africa (Cooper *et al*. 2009). The intervention began in the last trimester of pregnancy, and continued for 6 months postpartum. A total of 16 visits were delivered, particularly intensively in the first three postpartum months. The intervention consists of specific measures for encouraging mothers in sensitive, responsive, interactions with their infants. Findings were consistent with studies from HIC where home visiting support in pregnancy and postnatal, delivered by trained community workers, is associated with a range of improvements in maternal and child functioning (Olds *et al*. 2014). The fact that the intervention had a positive impact on both maternal sensitivity and infant security of attachment suggests that a long-term benefit can be anticipated. The same team has recently completed (with funding from Grand Challenges Canada Saving Brains programme) a long-term follow-up of the cohort when the children were 13 years old. These results will provide the first longitudinal data from an early intervention aimed at improving infant and child development in Africa.

The World Health Organization has estimated that 57 countries face health worker shortages; 36 of these countries are in sub-Saharan Africa (World Health Organization, 2006). Task shifting is one of the responses to the human resource crisis and is defined as the shifting or delegation of health services tasks from high level cadres of workers to lower level cadres (Zachariah *et al*. 2009). Scaling up early interventions, using community health workers within existing health systems (Cooper *et al*. 2002, Klein & Rye, 2004, Walker *et al*. 2006), will allow us to identify the best ways of integrating interventions into existing health platforms, while helping to achieve the desired outcomes related to targets set for child survival and well-being (United Nations, 2013). Data such as these would leverage better informed national and global policies for early child development. We also need to demonstrate social and economic impact – something that longitudinal lifespan research is well placed to show (Gertler *et al*. 2014). A coherent evidence base for scalable interventions that can be shown to have impact on economic development and human well-being is also essential (Lund *et al*. 2011). This is the language of most policy makers. Finally, and this is often forgotten, early interventions to improve infant and child development perhaps provide the most cost-effective means by which we can reduce the burden of adult mental illness.

### Resilience and differential susceptibility

#### Current context

Cooper *et al*. have shown how maternal depression and the endemic high levels of social adversity both have a significant adverse impact on maternal sensitivity (Cooper *et al*. 1999). However, the same team found that in this context almost two-thirds of the infants were securely attached at 18 months (Tomlinson *et al*. 2005). One of the reasons for the high rates of secure attachment in a context of extreme poverty was the protective contribution of social and cultural organisation that characterised this community. It was the features of the mother–infant relationship, postpartum depression and the mother's experience of partner support, that were the strongest predictors of infant attachment outcome (Tomlinson *et al*. 2005), rather than poverty *per se*. This in no way omits the role of poverty and adversity in contributing to increased levels of postpartum depression or the lack of partner support (poverty is instrumental in the genesis of both). Rather, these findings suggest that the simple measurement of socio-economic adversity is not, in itself, sufficient for a complex understanding of the factors involved in understanding parenting, poverty and infant and child developmental trajectory. Similarly, caution must be exercised in ascribing a causal role to parenting factors when in fact there are a number of other unmeasured confounders (such as inherited factors); unrecognised factors that we are not able to measure using available instruments; or even confounders that we are not aware of at all (Thapar & Rutter, 2009). The capacity of many infants and children to thrive, even in contexts of high adversity (resilience) requires substantial research focus.

#### Call for action: research on differential susceptibility to context: untangling average, hidden and factitious impact

It is no longer sufficient to view human development from a purely psychosocial or biological perspective. Rapid advances in developmental biology are providing an expanding number of techniques for tracking human development in its environmental context across all stages of the life cycle. Interactions between biology, psychology and environment have implications for individual outcomes and for evaluating the impact of early interventions (Belsky *et al*. 2013). A number of recent studies are providing evidence of how infants and children may be differentially susceptible to the environment or context (Bakermans-Kranenburg & Van Ijzendoorn, 2014). A commonly used metaphor to explain differential susceptibility is that of orchids and dandelions. Some infants and children are like orchids who with optimal early environments and context flourish, but do especially badly when they develop in sub-optimal environments. Conversely, there are infants and children who are like dandelions and who appear to be resilient when in adverse contexts, but do not benefit particularly from positive environments. There is new evidence of how if such interactions are ignored, measured impact frequently consists of an average of large impacts on susceptible children and small impacts on less susceptible children. Average findings conceal hidden impact and falsely portray factitious impact (Bakermans-Kranenburg & Van Ijzendoorn, 2014).

**Fig. 1.**
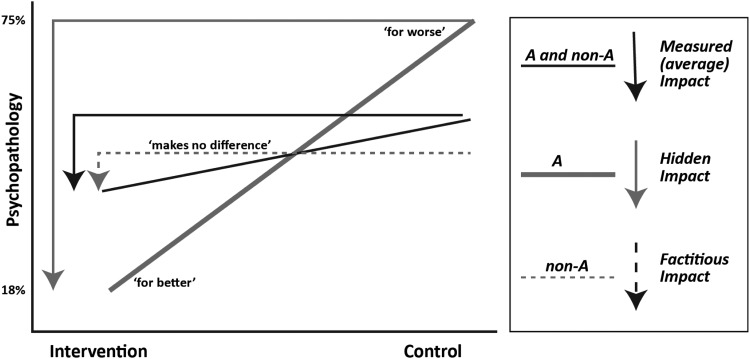
Differential susceptibility to environment.

Figure 1 uses an example from the Fast Track home visiting intervention to explain differential susceptibility. Fast Track was an intervention to prevent high-risk young children from developing externalising psychopathology (Bakermans-Kranenburg & Van Ijzendoorn, 2014). A purely psychosocial approach that measured externalising psychopathology at age 25 showed an intervention effect on reducing externalising psychopathology (black line). However, introducing individual differences in the glucocorticoid receptor gene (codes for the receptor to which the stress hormone cortisol binds) changes the picture completely. Individuals may carry either the A or non-A form of the gene. The Fast Track researchers had strong biological grounds for targeting this gene (Bakermans-Kranenburg & Van Ijzendoorn, 2014). They divided the same data used to construct the black line in Figure 1 into two separate data sets according to whether the individuals carried the A or non-A forms of the gene. Plotting this data yielded the grey line (A carriers) and the dotted line (non-A carriers). Seen in this way, the same externalising psychopathology results at age 25 clearly illustrate the differential susceptibility to context (in this case differential susceptibility to intervention) phenomenon. A allele carriers (grey) in the intervention group turn out ‘for better’ (only 18% have psychopathology) whereas control group A carriers turn out ‘for worse’ (75% have psychopathology). For non-A carriers (dotted line), belonging to the intervention or control group makes no difference (56% and 57% have psychopathology, respectively) (Bakermans-Kranenburg & Van Ijzendoorn, 2014).

Without genotyping, measured impact reflects the average impact on A and non-A carriers combined (black line), while the real impact is underestimated for A carriers (hidden impact) and overestimated for non-A carriers (factitious impact). Differential susceptibility allows the concept of resilience to be unpacked in some important respects. If resilience is a blossoming for the better in the midst of adversity, then the grey orchid line makes it quite clear that resilience is not an inner trait but one that is strongly context dependent with a costly downside when things turn out for worse. Or is resilience a quality of those who do not crash despite adversity – the 40% of dandelions in the Fast Track study who did not develop psychopathology irrespective of whether they were in the intervention or control group? Rather than attempt to answer these questions by reifying resilience as one outcome, differential susceptibility points towards the need to focus on the bigger picture of multiple interacting factors across multiple bio-psychosocial levels.

At the population level, differential susceptibility raises very real impact-evaluation and theory of change questions that are only beginning to come into focus, and will require significant interdisciplinary collaboration across the bio-psychosocial framework including inputs from economics, geography, sociology, public health and law. In order to expand the empirical base going forward, research in Africa should take steps towards integrating biological metrics and differential susceptibility moderators into the conception, design and implementation phases of their work (Shonkoff & Levitt, 2010). In sum, the longitudinal research priority that we are proposing for African infant mental health and child development research should employ biological metrics, differential susceptibility moderators as well as psychosocial metrics. If this is done, such an approach has the potential to become a potent tool for understanding infant and child development in Africa.

In this paper, we have outlined the current challenges to conducting research into infant mental health and child development in Africa. We have also proposed areas in which African research can be strengthened and in doing so have made a call for a series of actions that we believe will ensure that Africa is able to substantially contribute to the next wave of global infant mental health and developmental research in the next two decades.

## Footnotes

† The notes appear after the main text.

## Note

1 The word ‘infant’ means one who is unable to speak. The age range for what constitutes infancy is fluid, but for the purposes of this paper we will refer to the period of birth to the age of 3 years.
